# Poly Lactic Acid (PLA) Nanocomposites: Effect of Inorganic Nanoparticles Reinforcement on Its Performance and Food Packaging Applications

**DOI:** 10.3390/molecules26071967

**Published:** 2021-03-31

**Authors:** Mehrajfatema Zafar Mulla, Md Ramim Tanver Rahman, Begonya Marcos, Brijesh Tiwari, Shivani Pathania

**Affiliations:** 1 Food and Nutrition Program, Environment & Life Sciences Research Center, Kuwait Institute for Scientific Research, P.O. Box 24885, Safat 13109, Kuwait; mfmulla@kisr.edu.kw; 2 Faculty of Pharmacy and Institute of Nutrition and Functional Foods, Université Laval, Québec, QC G1V 0A6, Canada; ramimbau@gmail.com; 3 Laboratory of Medicinal Chemistry, CHU de Québec Research Centre, 2705 Boulevard Laurier, Québec, QC G1V 4G2, Canada; 4 IRTA, Food Quality and Technology, Finca Camps i Armet s/n, 17121 Monells, Spain; begonya.marcos@irta.cat; 5 Teagasc Food Research Centre, Food Chemistry and Technology Department, Ashtown, D15 KN3K Dublin, Ireland; 6 Teagasc Food Research Centre, Food Industry Development Department, Ashtown, D15 KN3K Dublin, Ireland; shivani.pathania@teagasc.ie

**Keywords:** poly lactic acid, PLA, nanoparticles, nanomaterials, nanocomposites, antimicrobial, degradation

## Abstract

Poly lactic acid (PLA) is a compostable, as well as recyclable, sustainable, versatile and environmentally friendly alternative, because the monomer of PLA-lactide (LA) is extracted from natural sources. PLA’s techno-functional properties are fairly similar to fossil-based polymers; however, in pristine state, its brittleness and delicacy during processing pose challenges to its potential exploitation in diverse food packaging applications. PLA is, therefore, re-engineered to improve its thermal, rheological, barrier and mechanical properties through nanoparticle (NP) reinforcement. This review summarises the studies on PLA-based nanocomposites (PLA NCs) developed by reinforcing inorganic metal/metallic oxide, graphite and silica-based nanoparticles (NPs) that exhibit remarkable improvement in terms of storage modulus, tensile strength, crystallinity, glass transition temperature (Tg) value, antimicrobial property and a decrease in water vapour and oxygen permeability when compared with the pristine PLA films. This review has also discussed the regulations around the use of metal oxide-based NPs in food packaging, PLA NC biodegradability and their applications in food systems. The industrial acceptance of NCs shows highly promising perspectives for the replacement of traditional petrochemical-based polymers currently being used for food packaging.

## 1. Introduction

The disposal of a huge quantity of plastic waste is one of the primary concerns and has led today’s consumers and environmentalists to demand a reduction in unnecessary plastic use in packaging applications. The Great Pacific plastic patch, marine litter and presence of microplastics in tap water have received a huge amount of media attention, rightly so. This waste issue can be well-tackled by the judicial use of sustainable resources to extract and/or derive bio-renewable polymers. Since 2015, the demand for a sustainable packaging solution has stimulated the innovations in biodegradable polymers for food packaging applications. The present enthusiasm for green innovations has led researchers to develop plant-based packaging for industrial packaging applications. Global production data suggests that the bioplastics production capacity is expected to increase from around 2.1 million tonnes in 2019 to 2.4 million tonnes in 2024 [1].

Plant-based biodegradable polymers can be categorized into two groups based on their availability: (1) natural plant polymers: Plants produce a number of natural polymers such as rubber (*cis*-1,4-polyisoprene), cellulose, starch, gluten (glutenin and gliadin), zein, soy protein isolate, gums, etc., and (2) from renewable resources: These polymers are derived from natural resources via processing, such as fermentation, ring opening polymerisation, etc., to form poly lactic acid (PLA), poly hydroxyl kanoates (PHAs), poly hydroxy butyrate (PHB), etc. It is also worthwhile to note that fossil sources can also be used to derive biodegradable polymers such as polybutylene adipate terephthalate (PBAT) and polycaprolactone (PCL); however, their discussion is beyond the scope of this review, and therefore, their properties and applications have not been discussed in this article.

**Figure 1:**
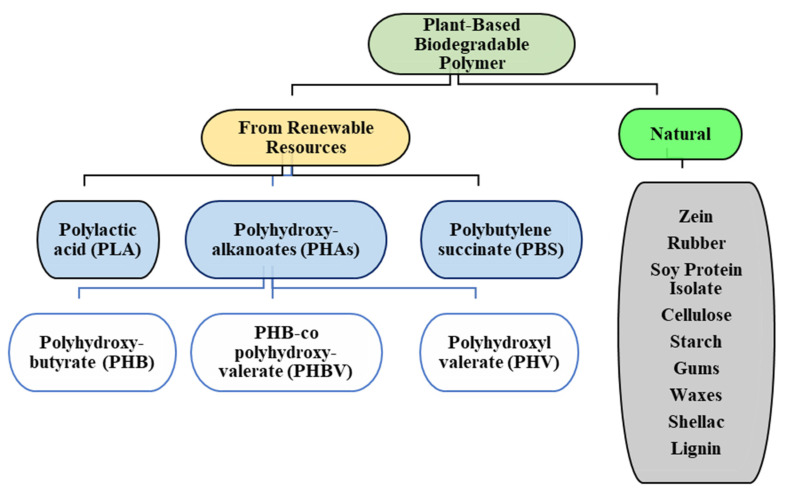
Different classes of plant-based biodegradable polymers.

This general classification of biodegradable polymers is illustrated in Figure 1.

Among several conventional biopolymers, PLA has been highly exploited commercially as a packaging material, as well as in interventions such as nanoparticle reinforcement to improve its properties for different packaging applications. PLA is produced from lactic acid through the fermentation of renewable resources, such as rice, wheat, corn, cane, potatoes, beets, etc. PLA is a linear aliphatic thermoplastic exhibiting semicrystalline, as well as amorphous, properties, possessing explicit clarity and achievable flexibility. In industrial production processes, three stereoisomeric forms of PLA can be synthesised, which are poly (L-lactide) (PLLA), poly (D-lactide) (PDLA) and poly (DL-lactide) (PDLLA).

It has been also classified as GRAS (generally recognised as safe) by the United States Food and Drug Administration (FDA). Moreover, it is produced from renewable resources, has no toxic effects and shows great potential in food and biomedical applications [2]. A life cycle analysis showed that PLA is more convenient than polyethylene terephthalate (PET) when the ultimate target of PLA bottles is recycling [3]. Studies have shown that the non-renewable energy use (NREU) and global warming potential (GWP) of PLA is superior compared to petrochemical-based plastics [4].

PLA has been widely accepted; however, in its original form, PLA as a polymer presents challenges such as breakability and fragility during thermoforming due to its inherent mechanical, barrier and thermal properties. In order to overcome this problem, nanoparticles (NPs) are reinforced in a PLA biopolymer. The concept behind reinforcement is the high surface area (As) and As-to-volume (Vs) ratio of nanoparticles that brings about a more noteworthy interfacial area and expanded synergy between the polymer chain and the nanoparticles; this expanded connection improves the properties of nanocomposites (NCs) [5]. NP reinforcement can improve the mechanical, thermal and antimicrobial properties, which is why several food industries are in the process of adopting bio-NC polymers for packaging applications. Several organic NPs derived from protein, peptides, lipid, polysaccharides, starch, chitosan, etc. can improve PLA properties to a certain extent; however, the derived NCs exhibit weak antimicrobial properties compared to their inorganic counterparts [6]. Inorganic NPs such as metals; metal oxides and minerals (Ag: silver, MgO: magnesium oxide, ZnO: zinc oxide, TiO_2_: titanium dioxide, Ag-Cu: silver-copper, HNTs: halloysite nanotubes, HA: hydroxyl apatite, silica, alumina, magnetite, zirconium oxide and CaCO_3_: calcium carbonate) have been known to considerably improve the properties of PLA-based films. These inorganic fillers have been reported to enhance PLA’s properties, such that these NCs can replace fossil-based polymers in various non-food and food applications. Based on these findings, the authors attempted to review the regulations and literature to evaluate, assess and document the research extent and their applications. There is abundant literature available on PLA-based polymers in the scientific databases [2,7]. The principal focus of this review is to elaborate PLA-based NCs reinforced with inorganic NPs—specifically, metal/metallic oxide, graphite and silica-based nanoparticles and their characteristics—as outlined in the scientific literature. This paper also discusses the regulations related to the reinforcement of inorganic NPs in PLA or biodegradable polymers and its usage as food contact materials and processing techniques used to date. Section 2 of this review discusses the rheological, mechanical, thermal, structural, barrier, morphological and antimicrobial properties. Section 3 outlines the effect of NP reinforcement on the biodegradability of PLA, and Section 4 lists documented applications of PLA NCs in fresh and processed food packaging. To the best of our knowledge, these findings, as presented in this review, have not been reviewed yet. We have extensively reviewed and presented the works carried out in this area during the last decade.

Methodology in search and review of literature: The guidelines of preferred reporting items for systematic reviews and meta-analyses (PRISMA) developed for systematic article review has been applied in the search and review of the literature for this article [8].

### 1.1. Search Strategy and Selection of Articles

A systematic search was carried out until 30th December 2020, with the help of the keywords “poly lactic acid”, “PLA”, “composites”, “metal”, “metal oxide”, “silica”, “graphite”, “inorganic”, “nanoparticles”, “food packaging”, “biodegradation”, “antimicrobial”, “extrusion”, “cast” and “film”, along with the Boolean operators AND, OR and NOT. Web of Science, Scopus database and Google scholar were used to accurately achieve the desired results. All the articles were read carefully, and the articles other than PLA and inorganic nanoparticles and food applications were removed. After a systematic review of the carefully chosen literature, the results of the authors were compared and considered until a consensus was reached.

### 1.2. Regulation Related to Inorganic Nanoparticles and Food Packaging Applications

Recently, the application of inorganic NPs in biodegradable food packaging have emerged as a breakthrough technology with dual benefits, such as improving the strength of materials and delivering antimicrobial properties. NPs of metal and metal oxides have been researched vigorously for their applications in food packaging, and, in particular, ZnO and TiO_2_ NPs have been widely used in food storage containers and food additives [9]. However, we observed differences, within countries, with regards to the regulations for the use of each NP and inorganic materials in food contact materials or matrices.

Zinc chloride, zinc gluconate, zinc oxide, zinc stearate and zinc sulphate are listed as GRAS materials (FDA, 2011) [10]. TiO_2_ is mainly used in the food industry as an additive E171 for whitening and texture improvement. It is present in several food products, such as cheese, cream, curd, chewing gum, candies, powdered sugar toppings, etc. TiO_2_ is an FDA-approved colouring additive with a regulated limit of its use up to 1% of the weight of the food. On the contrary, concerns have been raised on the usage of the E171 food additive, owing to its potential hazards with respect to humans and the lack of scientific evidence for its safety [11]. Most recently, silver nanoparticles (Ag NPs) are being used in many plastic food storage containers, plastic bags or boxes. However, considering the insufficient evaluation and testing of food containers, the United States Environmental Protection Agency (US EPA) prohibited the manufacturing, as well as trading, of food containers reinforced with silver NPs [12,13]. The permissible exposure limit recommended by the US National Institute for Occupational Safety and Health (NIOSH) is 0.01mg/m^3^ for all forms of silver [14]. Table 1 lists the regulations related to the use of inorganic materials or NPs as food contact materials.

Research work is also advancing towards ascertaining the safety of newly developed PLA NCs. Vasile et al. [15] studied the migration behaviour of NPs in stimulant media and found accepted values according to Regulation No. 10/2011 [16]. Pereiera et al. [17] also found similar results for PLA NCs. Their findings will be discussed in Section 5 of this review.

**Table 1:** Regulations and guidance related to inorganic substances and nanoparticles (NPs) reinforced in packaging materials as food contact materials. FDA: Food and Drug Administration TiO_2_: titanium oxide

NPs/Regulation	Description	SML (Specific Migration Limit)	Reference
TiO_2_/FDA	FDA-approved TiO_2_ is a component of coating agent Used as a preservative in latex emulsions at 2.2 PPM (based on silver ion concentration) in dry coatings irrespective of its size.		[18]
Zinc oxide	Food Contact Materials (FCM No. 1050). Permissibility as transparent ultraviolet light absorber in unplasticised polymers up to 2% by weight.	Zinc oxide, nanoparticles, does not migrate in nanoform when used in unplasticised polymers, and, therefore, safety evaluation should focus on the migration of soluble ionic zinc	[19]
Zinc/(Regulation (EU) No. 10/2011)	Additive for plastic materials and articles in contact with food expressed as zinc (FCM No. 402).	25 mg/kg food or food simulant	[19]
Aluminium/Regulation EU 10-2011/EU 2020/1245	Plastic materials and articles shall not release the aluminium in quantities exceeding the specific migration limits.	1 mg/kg food or food simulant	[20]
Copper/Regulation EU 10-2011/EU 2020/1245	Plastic materials and articles shall not release the copper in quantities exceeding the specific migration limits.	5 mg/kg food or food simulant	
Zinc/Regulation EU 10-2011/EU 2020/1245	Plastic materials and articles shall not release the Zinc in quantities exceeding the specific migration limits.	5 mg/kg food or food simulant	
Iron/Regulation EU 10-2011/EU 2020/1245	Plastic materials and articles shall not release the iron in quantities exceeding the specific migration limits.	48 mg/kg food or food simulant	
TiO_2_/EU 2020/1245	TiO_2_ surface treated with fluoride modified alumina (FCM No 1077) as an additive in plastic food contact materials.	Only to be used at up to 25.0% *w/w*, including in the nanoform.	
Montmorillonite clay modified with hexadecyltrimethylammonium bromide	Only to be used as additive at up to 4.0% *w/w* in polylactic acid plastics intended for long-term storage of water at ambient temperature or below.	The particles can form platelets in the nanoparticle range (<100 nanometres) that are not expected to migrate as they are oriented parallel to the plastic surface fully embedded in the polymer.	[21]
Titanium nitride, nanoparticles/Regulation EU 10-2011	In PET, the agglomerates have a diameter of 100–500 nm consisting of primary titanium nitride nanoparticles; primary particles have a diameter of approximately 20 nm.	No migration of titanium nitride nanoparticles. Only to be used in PET bottles up to 20 mg/kg.	
Graphite/Regulation EU 10-2011	It is not allowed to be used as monomer or other starting substance or macromolecule obtained from microbial fermentation and the migration results cannot be corrected by the Fat consumption reduction factor.	Listed in authorised additives without specific migration limit list. Not specified.	
Silver/European Commission reference number (Ref. No.) 86437	European Food Safety Authority (EFSA) recommended to set the limits for total permitted migration.	0.05 mg silver/kg food	[22]

PPM = Parts per million; PET = Polyethylene terephthalate; TiO_2_ = Titanium dioxide.

### 1.3. Reinforcement of Organic NPs for Development of PLA-Based Nanocomposites

Synthesis of PLA-based NCs reinforced with inorganic NPs include three major techniques: (1) in situ polymerisation, (2) solvent or solution casting and (3) melt mixing.

### 1.4. In Situ Polymerisation

The in-situ polymerisation technique involves the addition of an initiator (e.g., benzoyl peroxide or 2,2′-azobis (isobutyronitrile) in the presence of radiation or heat, followed by a series of polymerisation measures that results in the amalgamation of NPs and a polymer matrix. The process permits sufficient time for the formation of covalent linkages and, therefore, requires much less NPs to achieve better performances in NCs—for example, mechanical properties when compared with the melt extrusion technique [23,24]. Additionally, a high concentration of NPs in NCs is attained without initial exfoliation, unlike solution casting. This process is not restricted to developing NCs through various condensation reactions and covalent bond formations but, also, is used to form noncovalent-bonded composites [25]. The limitations of this method are the use of excess reagents in the polymerisation step.

With regards to PLA-based NCs, several examples of in situ polymerisation can be found in the literature. Recent advances in this technique include the elimination of the initiator, therefore extending the applications of the NCs developed from this process in food packaging. One such example is the synthesis of PLA NCs via the in-situ polymerisation of L-lactide with silane-modified nanosilica and montmorillonite without using an initiator where the ring opening of the epoxy group is favoured by moisture (present in the polymerisation apparatus) and lactic acid present in L-lactide, providing vicinal alcohol groups initiating the polymerisation of lactide [24].

### 1.5. Solvent or Solution Casting

It is one of the simplest methods of NCs preparation. The Principle of Stokes law is used in solution casting, wherein solvent selection plays a critical role in the overall success of this process. Solvent selection is based on the miscibility of the NPs and PLA monomers. A selected solvent is used to disperse NPs and PLA separately, as the dispersibility of the NPs and PLA varies depending on their molecular weight and surface properties. After the dissolution process is complete, both the solutions containing dispersed NPs and dissolved or swelled PLA matrix solutions are combined and mixed using ultrasonication or vigorous stirring for an interval of time. Further, this suspension is divided into moulds to allow composite formation, and finally, the solvent is removed from the system using a vacuum [26,27]. The critical control points during solution casting are the perfect solvent selection, comparative conditions between the solvent and polymer and the external environment conditions. Moreover, the complete drying of a solvent is essential for safety reasons, as well as NC performances [28].

PLA as a polymer demonstrates excellent solubility in organic solvents such as benzene, dimethyl formamide (DMF), tetrahydrofuran (THF), dioxane and chlorinated solvents. Most of the inorganic nanoparticles (metal, oxides and semiconductors) are hydrophobic, therefore exhibiting meagre dispersibility in polar solvents. The modification of the hydrophobic groups of NPs into a hydrophilic group via several treatments or oxidation can enhance their dispersion in solvents. Furthermore, ultrasonication can also be used to obtain rapid metastable dispersion of inorganic NPs in organic solvents. Several authors preferred the solution intercalation method for casting PLA-based NC films and observed an improvement in the thermal properties of films [29].

### 1.6. Melt Mixing

This is a physical method of NC formation. In this process, the NPs and polymer matrix are mixed at meld conditions with the help of a double-screw extruder followed by a single-screw extrusion to form films. Within the extruder, the transmitted heat blends the PLA matrix and NPs to form NCs. The developed NCs are passed through blow or cast lines using single-screw extruders to form films of varying strengths and properties. The significant advantage of this method over the other listed methods is that it is safe, environmentally friendly and cost-effective. Moreover, this method has enabled commercial vendors to produce films in bulk quantities [28]. Research studies have been carried out to develop PLA-based film doped with organic NPS such as MgO, TiO_2_, ZnO, etc. using the melt mixing process [30,31,32]. Limitations that restrict this method as compared to in situ polymerisation and solution casting are severe conditions that can damage the NPs or PLA matrix, in addition to an inadequate ability of NPs to produce proper dispersion [33].

## 2. Characterisation of PLA-Based NCs Reinforced with Inorganic NPs

### 2.1. Rheological and Mechanical Properties

Pristine PLA is a pseudoplastic and non-Newtonian fluid. PLA is also characterised by its property in exhibiting a plateau in Newtonian behaviour at a lower frequency range. Basic micro- and mesoscopic configurations of polymer chains can be meticulously explored by virtue of rheological experiments as an elastic modulus (G′), viscous modulus (G′′) and complex viscosity (η*). Thermoplastic materials’ seamless performances during processing operations are paramount for industrial operations, and this attribute can be estimated by studying the rheological properties of melts [34].

It has been established that NP reinforcement can improve the G′ and G′′ values of PLA NCs. Zhang et al. [35] studied the rheological properties of extruded PLA/TiO_2_ NCs and compared them to a neat PLA matrix. They found that storage modulus G′ showed greater increments at higher TiO_2_; however, at a lower frequency region, the presence of TiO_2_ showed a minor increase in viscous modulus G′′. In another study, a monotonic rise of G′ (at a selected frequency region) was observed in a PLA matrix loaded with clay (3–9%), while the rise in G′′ was similar to a pristine PLA matrix specifically at a greater frequency region [36]. PLA/graphite NCs also showed higher G′ and G′′ values specifically at lower frequency ranges, which would have superimposed adequately onto the increased relaxation time. Moreover, a strident increase (a count of more than three magnitudes) in the G′ value was observed for a PLA/graphite composite, which meant an improvement in the elasticity of the polymer melt [37].

The η* value can effectively determine the performance of the polymer in the composite film formation during extrusion processing, and a lower value would usually indicate a hindrance in the composite formation. For PLA, the η* value demonstrates a minor dependence on the frequency. However, the reinforcement of NPs affects the η* value of the PLA matrix, which is determined by the type of NP added to the polymer. The low loading content of TiO_2_ (0.5 wt% and 1 wt%) increases the η* value of the NC melt [35], whereas a significant decrease in the η* value and shear thinning behaviour was exhibited (regardless of the grain magnitude) as ZnO was reinforced at a loading concentration of 4 wt%. The reinforcement of ZnO in the PLA matrix led to a reduction in the molecular weight and catalytic depolymerisation of PLA, causing a decrease in the η* value as a function of the frequency [37]. An analogous drop in the η* value with the augmented frequency value was evident for the Ag–Cu-reinforced PLA/PEG matrix; however, ZnO does not seem to follow the same direction.

The presence of graphite nanoparticles improved the η* value at both the lower, as well as higher, frequencies as compared to the control PLA. Graphite nanoparticles are miscible, which improves the physical crosslinking of molecules and significantly enhances the firmness of the molecular chain, further improving its mobility resistance. However, at the low shear rate region, an augmented shear stress and additional time was taken by the NC melt to flow. This could be due to the impeded molecular chain movement owing to the crisscrossing structure of the nano graphite. A greater shear thinning behaviour was depicted for PLA/graphite NC at a higher shear rate, which could be employed to the preferably symmetric alignment of nano graphite fragments, resulting in impairment of the crisscrossing structure of the nano graphite. This shows how the molecular chain movement was hindered and also clarifies the lattice formation of the nano graphite [38].

Mechanical properties: Tensile stress (TS), Elongation At Break (EAB) and the tensile modulus (also recognised as Young’s modulus) of bio-NCs are the mechanical properties that are considered to determine the applications for biopolymers (Table 2). Highly ordered molecular structural forms, e.g., semicrystalline PLA, are mostly preferred for elevated mechanical properties such as tensile strength and the elastic modulus. The tensile strength and Young’s modulus of the pristine PLA range from 40 to 70 MPa and 1 to 3 GPa, respectively. Contrary to the studies published in the previous decade, mainly on the reinforcement of clay NPs in the PLA matrix, this decade’s scientific publications were focused towards improving the mechanical properties of the PLA matrix with the help of new formulations such as organic NPs (metal oxides and graphite).

PLA NC films reinforced with montmorillonite (MMT) clays illustrated a drop in TS values that could be because of the uneven dispersion of organoclays in the polymer structure. Interestingly, the PLA/Cloisite 20A NC exhibited an increase in mechanical properties [39]. In contrast to the aforementioned observations, a PLA matrix reinforced with inorganic NPs depicted a different scenario, where it was more concentration-dependent for the improvement of the composites’ mechanical properties. The addition of Ag-NPs at 1 wt% had no significant effect on the mechanical properties of PLA [40]. Interestingly, the loading of TiO_2_ up to 2 wt% and Halloysite nanotubes (HNTs) up to 6% showed an improvement in TS while further increases in the loading dropped the TS value compared to neat PLA [33,35]. An improvement in the tensile stress was also observed due to 5 wt% nonographite reinforcement in the PLA matrix [38]. Similar findings were obtained for NCs reinforced with metal oxides, such as ZnO and MgO, by Ghozali et al. [41]. The increase in TS was accompanied by a restriction of the chain movement, which leads to abundant PLA. NP synergy causes a greater shift of stress from the matrix to NPs under pressurised conditions. The deprivation of the polymer matrix was ascribed to unfolding/unzipping. Moreover, the reinforcement of untreated metal oxide NPs into PLA led to transesterification reactions during extrusion processing, which could also be one of the reasons for a lower TS value at a higher loading concentration. Silanes treated with ZnO were used to overcome this issue and observed an improved tensile strength up to a loading concentration of 2%, and a further increase in the concentration of ZnO dropped the tensile stress, which could be due to the diminution of the molecular weights and formation of low molecular products [42]. These limitations were further improved by Arfat et al. [43]; their group reinforced the PLA film with surface-treated ZnO by 3-methacryloxypropyltrimethoxysilane and, as a result, a significant effect on the TS and EAB values up to 10% ZnO loading concentration. This was attributed to the hydrogen bonding between the PLA hydroxyl group and siloxane groups formed due to the surface treatment. The incorporation of the GO–ZnO (graphene oxide–zinc oxide) nanohybrid improved (14.2%) the tensile strength of PLA, which could be related to the greater physical or chemical crosslinking among the planar geometry of GO–ZnO and the PLA matrix [44]. In particular, De Silva et al. [45] further enhanced the effect of ZnO and HNTs by depositing ZnO on HNTs, which effectively improved the ductility and tensile strength of PLA compared to the PLA–ZnO composite films; these changes are understood upon the presence of the tubular shape of HNTs, which efficiently transfer the stress and improve the interfacial interaction and dispersion. The EAB value of PLA increased up to 2 wt% TiO_2_ loading, whereas there was no significant difference in the EAB value due to the reinforcement of the ZnO (1–3%) and GO–ZnO (0.2–1.0%) nanohybrids. Moreover, the addition of HNTs up to 3% and ZnO up to 1% was reported to possess improvements in the Young’s modulus or rigidity, and further increments in the loading concentration showed no significant changes in the Young’s modulus; this could have resulted from the aggregation of NTs/NPs at a higher concentration, while the improvement in rigidity resulted from the hydrogen bonding between PLA hydroxyl group and siloxane groups of the HNTs.

**Table 2:** The effects of inorganic NPs on the mechanical and thermal properties of poly lactic acid (PLA)-based nanocomposites (NCs).

Films	Mechanical Properties			Thermal Properties				Reference
	TS (MPa)	EAB (%)	Young’s Modulus (GPa)	Tc (°C)	Tm (°C)	Tg (°C)	Xc/%	
Neat PLA	-	-	2.05	-	170	-	10.2	[33]
PLA–% HNT(QM)	-	-	2.50	-	167	-	15.1	
PLA–6% HNT(QM)	70	-	2.70	-	163	-	6	
PLA–12% HNT(QM)	-	-	2.80	-	162	-	6.6	
PLA	42	9.3	2.70	108	169	64	6.6	[42]
PLA–0.5% ZnO	-	-	-	110	170	65	3	
PLA–1% ZnO	41	13	2.90	111	171	64	2.8	
PLA–2% ZnO	39	7.1	3.00	110	171	64	3	
PLA–3% ZnO	35	12.9	2.80	111	172	63	2.8	
PLA	60.0	8.4	-	-	-	65.45	-	[44]
PLA/0.2 wt% GO–ZnO	67.7	6.1	-	-	-	70.82	-	
PLA/0.5 wt% GO–ZnO	68.5	5.7	-	-	-	70.90	-	
PLA/1.0 wt%GO–ZnO	72.3	6.3	-	-	-	69.24	-	
PLA	59.8	11.6	2.3	100.5	169.1	-	6.51	[35]
PLA–0.5 wt% TiO_2_	61.7	12.9	2.4	106.1	169.5	-	6.63	
PLA–1 wt% TiO_2_	61.5	19.1	2.4	106.5	169.5	-	5.91	
PLA–2 wt% TiO_2_	60.7	24.0	2.3	104.5	170.0	-	5.84	
PLA–5 wt% TiO_2_	57.6	13.7	2.4	100.4	169.0	-	5.47	
PLA–10 wt% TiO_2_	55.9	13.7	2.3	98.6	168.5	-	4.83	
PLA–15 wt% TiO_2_	50.3	10.4	2.8	99.0	169.3	-	4.67	
PLA	69.28	2.14	4.0	119.2	148.7	57.5	10.1	[15]
PLA/ZnO:Cu/Ag 0	44.81	3.30	2.9	95.4	149.8	44.0	30.8	
PLA/ZnO:Cu/Ag 0.5	45.32	2.78	2.9	98.3	149.5	46.4	31.2	
PLA/ZnO:Cu/Ag 1	48.39	2.67	3.1	99.1	149.7	48.5	31.8	
PLA/ZnO:Cu/Ag 1.5	47.28	2.61	3.0	98.7	149.3	47.5	31.7	
PLA	65.3	-	-	115.97	153.63	60.25	25.93	[46]
PUHA	55.2	-	-	112.99	155.83	62.64	30.66	
PMHA	68.8	-	-	108.68	156.56	63.23	37.01	
Neat PLA	29.1	4.4	1.89	105.77	146.56	58.09	-	[30]
PLA/1 wt% MgO	34.0	3.3	2.41	110.64	145.71	57.34	-	
PLA/2 wt% MgO	37.5	3.9	2.47	120.93	148.19	57.98	-	
PLA/3 wt% MgO	26.6	2.3	2.10	114.90	147.73	57.29	-	
PLA/4 wt% MgO	26.2	2.4	1.96	113.57	146.86	57.53	-	

HA = Hydroxyapatite; PUHA = PLA composite containing 10 wt% unmodified HA; PMHA = PLA composite containing 10 wt% modified HA; HNT = Halloysite nanotubes; PLA = Poly lactic acid; GO = Graphene oxide; TiO_2_ = Titanium dioxide; ZnO = Zinc oxide; MgO = Magnesium oxide; Ag = Silver; Cu = copper; TS = Tensile stress; EAB = Elongation At Break; Tc = Crystallization temperature; Tg = Glass transition temperature; Tm = Melting temperature; Xc = Degree of crystallinity; MPa = Megapascal; GPa = Gigapascal.

### 2.2. Thermal Properties of PLA-Based NCs Reinforced with Inorganic NPs

The thermal analysis of bio-NCs is an important attribute in establishing food packaging applications. Differential scanning calorimetry (DSC) and thermogravimetric analysis (TGA) explicitly reveal the melting, crystallisation and corresponding enthalpy and entropy changes, as well as the characterisation of the glass transition and thermal degradation behaviours, of the biopolymers. Crystallinities depict the proportion of the crystalline region in comparison with the amorphous region. The crystallinity of the PLA matrix varies according to the processing conditions, type of plasticisers and nucleating agents. The modulation of rheological, mechanical and thermal properties of PLA depends on its glass transition temperature, i.e., Tg. Therefore, Tg is most important parameter to envisage the PLA behaviour during processing, as a remarkable modification in the polymer chain mobility of PLA usually arises at or above the Tg level [2]. The thermal analysis or calorimetric parameters obtained by the DSC analysis of PLA NCs doped with NPs is listed in Table 2. The DSC traces revealed no significant changes in the Tg values for HNTs (up to 12%), which fluctuated from 61 to 63 °C, while there was slight decline in the Tm (melting temperature) and Tc (crystallisation temperature) values due to the reinforcement of NTs, which could be due to the HNT limited nucleation during PLA crystallisation [33].

Similar findings were obtained for PLA reinforced with ZnO (0.5 to 3%) and Ag (0.12 to 1.74%); one advantage over the addition of Ag NPs was an improvement in the crystallinity of the resulting NCs [42,47].

The reinforcement of MgO NPs did not significantly alter the Tg and crystallinity of PLA NCs [30]. The Tg of NCs is mostly influenced by the molecular weight, crosslinking, interaction between molecules and molecular chain flexibility. Later, a significant improvement in the Tg value was observed when PLA was reinforced with the GO–ZnO (0.2–1%) nanohybrid; it is hypothesised that a strong interfacial action occurred in favour of a large surface area of GO sheets and a reinforcing effect of ZnO impregnated on GO sheets, which further reduced the polymer chain mobility [44]. Vasile et al. [15] enhanced the thermal properties of PLA by reinforcing Cu-doped ZnO nanoparticles functionalised with Ag nanoparticles. PLA is practically amorphous as such due to the lower rate of crystallisation, which impact its utilisation in the automatic form fill machines used generally for packaging food items. Most of the selected nanoparticles showed a marginal effect on the crystallinity of the PLA matrix, while the ZnO:Cu/Ag nanoparticle reinforcement showed an increase in the Tc and Tg. A further improvement in crystallinity up to 31.8% as compared to pristine PLA was observed due to the effective heterogeneous nucleation that occurred due to the presence of extra nucleation sites (Ag and Cu). A similar increase in the Tg and Tm values was observed when ZnO or the Ag-Cu alloy was incorporated in PLA-PEG (polyethylene glycol)-based NCs. The incorporation of ZnO or the Ag-Cu alloy also improved the crystallinity of NCs, albeit the Ag-Cu alloy depicted lower nucleating properties than ZnO [37]. The Tg values of PLA NCs reinforced with specific silane-treated ZnO and untreated ZnO showed no more improvements, which could be a result of the small amount of NP reinforcements [42,48,49]. However, a recent work by Arfat et al. [43] detected an increase in the crystallinity of PLA-PEG NCs by the incorporation of surface-treated ZnO by 3-methacryloxypropyltrimethoxysilane; these results related to the improved nucleation carried out due to a greater concentration of NP reinforcements. In addition, the effect of propanoic acid functionalised TiO_2_ nanoparticles on the PLA matrix and observed a greater degree of crystallinity for functionalised nanoparticle-loaded NCs, which further observed an intermittent decrease of crystallinity for NCs containing a higher content of TiO_2_ (more than 2%); this suggests that crystallisation happened in the NCs during both treatments. Functionalised TiO_2_-reinforced NCs depicted lower Tg values as compared to NCs containing untreated TiO_2_, while both the NCs showed a greater Tg than pristine PLA; this indicates a greater withdrawal of the polymer chain mobility in NCs containing untreated TiO_2_ NPs [50]. Moreover, there is an effect due to the ZnO NP size (100 and 50nm) on PLA-PEG-based NCs, and we observed improved Tg values for NCs incorporated with 100nm for all the chosen NP concentrations, excluding 0.5 w% [43]. Further, an amorphous crystal structure was observed for PLA/MgO composites that showed no crystalline phase due to the reinforcement of MgO [30].

To gain insight into the thermal stability and bond dissociation of different molecules of NCs, TGA has been frequently deployed by researchers. Alakrach et al. [51] studied the effects of different HNT nanotubes on PLA/HNT NCs and observed several thermal events, including weight loss, which occurs at three distinguishable regions. The first weight loss near or below 100 °C occurs due to free water loss, the second weight loss between 200 °C and 400 °C occurs due to a loss of the hydration water molecule between HNT and PLA and the third weight loss at temperatures greater than 400 occurs due to dehydroxylation. It is noteworthy that the thermal degradation temperature (Td) of PLA/HNTs is slightly lower than neat PLA, which is due to the release of the entrapped water molecules from coiled walls of HNTs, where HNTs possess a lower aspect ratio. Furthermore, at a greater concentration of HNTs, the 50% weight loss temperature for the NCs also increased compared to the pristine PLA. This improvement in the thermal stability of the NCs in the presence of HNTs could be due to bound degraded products in the lacuna of HNTs and the char residue formation, which might lower the diffusion of volatile losses through its insulating effect towards high heat [46]. An enhanced thermal stability for the PLA/ZnO composite was attributed to an excision and congregation of the ZnO on the surface of the NC films, which acts as shielding for the exterior of the substance; further, the ebullition of gas might even be deferred via the network effect of ZnO layers in NCs [52]. However, it is not the same with PLA NCs reinforced with silver nanowire (Ag NWs), where no significant change in the degradation temperature due to the addition of Ag NWs was observed; it could be a consequence of the lack of chemical and surface interactions between PLA and Ag NWs [47]. Ghozali et al. [41] observed a greater thermal stability for PLA/TiO_2_ NCs than PLA/ZnO and PLA/MgO and stated that it could be a result of the heat insulator property of TiO_2_ at the early thermal decomposition stage. These observations corroborate the results obtained by Ahmed et al. [48] for PLA/PEG/ZnO films. Further, these authors observed an enhanced thermal stability for the PLA/PEG film reinforced with Ag-Cu alloy, owing to the strong interaction between NPs (Ag-Cu alloy) and the polymer matrix and the elevated thermal stability of Ag-Cu alloy NPs. Liu et al. [53] also observed a change in the thermal stability of PLA/MO NCs owing to metal oxide doping (Bi_2_O_3_, CuO and Fe_2_O_3_) in the PLA matrix, and the trend for catalytic properties was Bi2O_3_ > Fe_2_O_3_ > CuO. The effect of surface-modified hydroxyapatite (HA) on an injection-moulded PLA matrix demonstrated an elevated thermal stability for PMHA (PLA reinforced with 10 wt% modified HA) owing to the improved hydrogen bonding of HA and PLA as a consequence of the abundant active hydroxyl groups and uniform dispersion of HA, which further efficiently disseminated the thermal energy over numerous bonds [46].

### 2.3. Structural/Spectral Properties of PLA-Based NCs Reinforced with Inorganic NPs

A Fourier-transform infrared spectroscopy (FTIR) analysis of the NCs is used to characterise the modifications made in the polymer matrix by studying the vibrational shift or change in intensity of the major IR bands owing to the incorporation of NPs. Previous reports on ZnO reinforcement in the PLA matrix revealed an increase in intensity of all the observed bands for the PLA matrix [52]. Shankar et al. [49] reported an increase in intensity of the band at 3654 cm^−1^ due to a weak secondary bond formation between the matrix and NPs. Recent reports revealed nonsignificant variations in the PLA bands when compared with metal oxide (MgO, ZnO and TiO_2_) and Ag-Cu-reinforced PLA. This could be associated with the lower amount of nanofillers used to produce the composite films exhibiting only surface-accessible or superficial interactions between the matrix and NPs rather than chemical interactions [30,37,41]. Moreover, the reinforcement of Ag NPs was linked with broadening of the band at 3493 cm^−1^ and was postulated to the van der Waals interactions between the hydroxyl functional groups of matrices and the higher electric potential of Ag-NPs [54]. It is noteworthy that the reinforcement of 0.5 wt% TiO_2_ (functionalised with propanoic acid) NPs in the PLA matrix displayed the commencement of a new band at 2926 cm^−1^, which was correlated to the CH_2_ asymmetric stretch of propanoic acid. PLA/HA composites imbibed with modified HA observed a shift of the peak at 3466 cm^−1^ to a lower region at 3455 cm^−1^, which depicted a hydrogen bond formation between the C=O groups of PLA and –OH groups of the modified HA. Moreover, a shift of the band at 1750 cm^−1^ to a lower region was also observed in composites reinforced with modified and unmodified HA. The presence of an additional −OH group for hydrogen bonding and an esterification reaction between these −OH groups and the terminal –COOH groups of PLA could be one of the possible reasons for this kind of shift [46].

X-Ray Diffraction (XRD): The XRD analysis relates to the exploration of the diffraction properties of the matrix in terms of their crystal structure and degree of crystallisation. All the PLA-based composites exhibited a strong peak at 2θ of ~16.5° ascribed to (200) and/or (110) planes of the typical rhombic crystal, indicating an entirely amorphous phase of the PLA films. Further, it was observed that the Ag NP reinforcements depicted five 2θ° of ~ 38.18°, 44.3°, 64.55°, 77.54°, and 81.71° peaks in addition to the broad PLA peak, which corresponds to 111, 200, 220, 311, and 222 crystallographic planes of face-centred cubic (fcc) silver crystals. Moreover, the intensities of these peaks were linearly related to the concentration of Ag NPs, which led to the hypothesis that Ag NPs are systematically imbibed in the PLA matrix [54]. Conversely, a typical crystallisation peak of PLA 2θ ~16.5° was visible only in pristine PLA and NCs containing less amounts of ZnO (0.5%). Further, NCs also depicted peaks at 2θ° of 31.6° and 2θ ~36.2°, corresponding to the diffraction planes of (100) and (101) of the crystalline form of ZnO, respectively [42]. However, PLA/ZnO composites at 2θ° of ~32°, 34°, 36°, 48°, and 58° were analogous to the crystal planes (100), (002), (101), and (102) of the PLA and (110) for the ZnO crystal structure. They further observed improvements in the intensities of the peaks at greater concentration of NPs, and the peak position was same at all the studied concentrations, which depicted the proper distribution of NPs without the layer formation of NPs in the matrix [52]. Shankar et al. [49] observed clear diffraction peaks in the range of 30–40° for the PLA/ZnO NPs composite films. It is further noted that the surface treatment of ZnO with 3-methacryloxypropyltrimethoxysilane revealed no significant change in the peak position of PLA and ZnO, suggesting that the crystalline property of NCs is well-preserved [43]. Interestingly, an amorphous crystal structure was observed for PLA/MgO composites at 2θ of ~16.5° and the crystalline structure of MgO at 2θ° of 36.78, 42.76°, and 62.16°, and these peaks depicted greater intensity at higher loading concentrations; further, there was no shifts in the peak position of the composites, which revealed the proper mixing of NPs in the PLA matrix [30]. Similarly, an increase in the diffraction intensities with an increase in the concentration of ZnO:Cu/Ag NPs in the PLA/ZnO:Cu/Ag composites was also observed [15].

### 2.4. Barrier Properties of PLA-Based NCs Reinforced with Inorganic NPs

One of the principal aspects of food packaging materials is their permeability and barrier efficacy towards the transmission of water vapour, gases, and aroma molecules. A high water vapour transmission rate (WVTR) and oxygen transmission rate (OTR) of the films significantly curbs the usage of a selected film as a prospective material for foodstuff packaging. PLA-based films exhibit high WVTR; therefore, the NP reinforcement strategy has been known to improve the barrier properties of PLA-based films. Fortunati et al. [55] observed a 4% reduction in the WVTR of NCs due to the impregnation of Ag (1%) NPs (Table 3). However, recent studies on ZnO NP impregnation in the PLA matrix reported a significant reduction in WVTR up to 9% of the loading concentration, while further increasing the concentration of NPs lowered the WVTR of NCs [49,56,57]. It was hypothesised that, firstly, the amorphous and, then, the crystalline phase of the matrix gets filled with NPs, which hinders the water vapour transmission through the tortuous path, resulting in an increased water barrier property. Further, a higher concentration of NPs can cause a drop in the water barrier property, which is attributable to the PLA matrix disruption, owing to disproportionate crowding of NPs in the dense crystalline region of the PLA matrix. Additionally, the intrinsic hydrophilic property of ZnO NPs can also heighten the WVTR of the NCs.

The silane treatment was effective in decreasing the WVTR at a lower concentration of ZnO NP impregnation due to the uniform distribution of NPs in the PLA matrix [43]. Clays can also provide gas and water vapour resistance and increase the biopolymer’s mechanical strength [58]. The reinforcement of TiO_2_ and HNT NPs revealed a unique trend parallel to a greater hydroxyl groups on the surface of the NPs, enhancing the mass transfer of water molecules through the matrix, thereby increasing the WVTR.

The gas permeation properties of polymer-based films are studied carefully in order to ascertain their potential use in the packaging of different food products. Some food products such as orange juice and dried and powdered food products are sensitive to external environmental conditions such as oxygen and, therefore, cannot be packaged in a gas-permeable film, as it will fail to perform its function to protect the enclosed food product. Plain PLA films are characterised by their inherent brittleness, which is generally treated by a plasticiser addition; however, their inclusion tends to lower the gas barrier properties of polymers. In order to offset the effects of plasticisers on the oxygen permeability, Cabedo and others [59] reinforced chemically modified kaolinite in amorphous PLA to form a kaolinite NC, which led to an increase in the oxygen barrier properties by about 50% [59,60].

The oxygen permeability showed a 22% reduction with a reinforcement of Ag and MgO (1 wt%) in the PLA matrix [30,55]. The decrease in the OTR was evident until a reinforcement concentration of 2%, while further loading enhanced the oxygen permeability that is attributable to the free spaces created at the borders of clustered MgO/ZnO NPs and the PLA matrix; these allow the easy passage of gaseous molecules and the creation of a path for permeation relative to pristine PLA. Similarly, the impregnation of ZnO NPs also reduced the OTR up to a 9 wt% loading concentration, and further loading increased the oxygen transmission. Moreover, Arfat et al. [43] treated ZnO NPs with 3- methacryloxypropyltrimethoxysilane and prepared a plasticised NC with the help of PLA and PEG and observed a drastic (55%) drop in the OTR value for NCs imbibed with treated ZnO up to a 10% loading concentration. NCs loaded with untreated ZnO also showed a decrease in OTR, but it was less as compared to NCs loaded with treated ZnO. It could be a result of the untreated surface of ZnO permitting a poor distribution of NPs in the matrix as compared to surface-treated NPs.

### 2.5. Morphological Properties of PLA-Based NCs Reinforced with Inorganic NPs

Scanning electron microscopy (SEM) is one of the amplification tools that has been used by several studies to trace the nanoparticle positionings in the NCs to trace the miscibility of NPs. In the films developed using the solvent casting process, a consolidated, uniform, and frictionless side of the film depicts a higher affinity and improved blending between the PLA matrix and solvent. The PLA film reinforced with metal oxides (TiO_2_, MgO, and ZnO) exhibited dissemination in the film and further caused crevice structures and the impression of pits on the film surface. Moreover, at a greater concentration of ZnO reinforcement, the crevice surfaces of films became cragged, and the clusters of major and minor dimensions are visible on the film. It could be attributable to inadequate interface between the PLA matrix and metal oxides. Previous reports on ZnO NPs reinforcement in the PLA matrix indicate a shortage of the surface treatment of ZnO NPs scattered in the solvent arrangement brought about by the appearance of the ZnO NPs on the outside of the film owing to an agglomeration of NPs [42,57]. This observation is linked with the concentration of NPs in the matrix; as the measure of NPs inside the composites expands, the level of agglomeration increases. These agglomerations can act as a weak point for the mechanical strength of NCs by reducing the stress encumbered. The surface treatment of NPs can elevate the concentration of –OH groups available for bonding with PLA during blending, which, in turn, can improve the diffusion of NPs in the PLA framework, eventually improving the mechanical property of the NC [46]. The visible agglomerate on the PLA matrix with greater and smaller aspect ratios for untreated TiO_2_ NP-reinforced NCs were observed to be the delicate agglomerates predominantly brought about by van der Waals powers [50]. Ultrasound treatment or dispersions have been employed to eliminate such types of agglomerations during film formation. Ultrasounds can break the intermolecular interactions and break up softer particle agglomerates. It has also been documented that agglomerates in the micrometre size range are hard due to a strong chemical bond formation that can resist breaking down [61]. The surface treatment of ZnO with 3-methacryloxypropyltrimethoxysilane enabled the better circulation and scattering of the NPs in the PLA framework [43,49]. These observations are consistent with the NCs developed by the reinforcement of surface functionalised (propionic acid) TiO_2_ NPs.

The coating or deposition of NPs on polymer surface using different techniques have been also studied by researchers for the uniform deposition of NPs. Doganay et al. [47] observed that coating Ag NWs with polyvinylpyrrolidone (PVP) showed stable dispersion at a higher concentration, where greater a NW-NW intersection barrier is built by the PVP layer on the lateral surface of the NWs. Several methods such as magnetron sputtering, plasma-enhanced chemical vapour deposition, and atomic layer deposition (ALD) have been studied. The nanometre scale atomic layer deposition of metal oxide layers has shown their feasibility as high-quality diffusion barriers on flexible PLA-based packaging materials [62]. This excellent conformity may give ALD a great advantage over the other coating or deposition techniques for future works.

### 2.6. Antimicrobial Properties of PLA-Based NCs Reinforced with Inorganic NPs 

Most metal oxide nanoparticles exhibit bactericidal properties through the generation of reactive oxygen species (ROS), although some are effective due to their physical structures and release of metal ions. Several investigations are documented as the use of organic/inorganic (metal) nanoparticles as antimicrobial agents against Gram-positive and Gram-negative bacteria. These materials’ intrinsic biological properties depend on several factors, depending on the nanoparticles involved: particle size, structure, and surface area. All possible combinations can show a higher antibacterial activity. Nisar et al. [63] and Sanchez-Lopez et al. [64] explained that electrostatic interactions are responsible for this antimicrobial activity, as positively charged metal NPs are attracted to negatively charged bacterial cell walls, resulting in disruption and consequently increasing their permeability. Further, these NPs induce the production of reactive oxygen species (ROS) inside the cell, which can disrupt the biological processes of bacteria and/or interact with cellular structures (e.g., proteins, membranes, and DNA), leading to bacterial death [65,66]. Most antimicrobial NC studies have focused on food packaging, and biocidal activity has always been targeted on the same bacteria, and mixed cultured bacteria have not been studied yet. It is also not certain if the bacteria become resistant to biocidal nanoparticles, as observed in the case of drugs. Interestingly, such antimicrobial properties can slightly delay the biodegradation process, and specific bacteria may not be involved in the biodegradation process, as they are susceptible to NPs. Inorganic NCs are currently developed to control or prevent microbial colonisation; this is being achieved by incorporating the polymer matrix with nanoparticles exhibiting a known antibacterial activity.

Several studies have reported the antimicrobial activity of NCs incorporated with inorganic NPs in PLA, such as ZnO against *Escherichia coli* and *Staphylococcus aureus* [44]; silanised ZnO against *Listeria monocytogenes* and *Salmonella enterica typhimurium* [43]; ZnO-doped silica NPs against *Escherichia coli* [65]; ZnO:Cu/Ag against *S. aureus* and *Pseudomonas aeruginosa* [15]; bimetallic silver–copper (Ag–Cu) nanoparticles with cinnamon essential oil against *Campylobacter jejuni, Listeria monocytogenes*, and *Salmonella typhimurium* [66]; TiO_2_ against *E. coli* and *Aspergillus fumigatus* [67]; TiO_2_/Lycopene pigments against *E. coli* and *S. aureus* [68]; and MgO against *E. coli* [30].

Antimicrobial properties are also influenced by the microbial attachment to the film surfaces, which is further affected by the material surface characteristics and type of microorganism. Smooth surfaces do not favour microbial adhesion and biofilm deposition, whereas rough surfaces have a greater surface area and may provide more favourable sites for colonisation. Moreover, microorganisms can adhere and colonise the porous surface preferentially, depending on the hydrophobicity and the surface charge of the NCs [69]. The adhesion process of microorganisms to the surfaces includes interactions, such as van der Waals, Lewis acid bases, and hydrophobic and electrostatic interactions [70]. PLA reinforced with ZnO:Cu/Ag NPs showed a better adherence against *S. aureus*, while inhibiting the growth of *P. aeruginosa*. The better adhesion to PLA NCs is related to the electrostatic affinity of Gram-positive bacteria. These bacteria have more peptidoglycan (negatively charged) than Gram-negative bacteria; thus, they adhere strongly to positively charged Zn and Cu ions in PLA NC, which explains the antimicrobial property of these NCs as compared to the plain PLA polymer [15].

## 3. Degradation Mechanism of Plant-Based Biodegradable Polymer PLA Reinforced with Inorganic Nanoparticles

**Figure 2:**
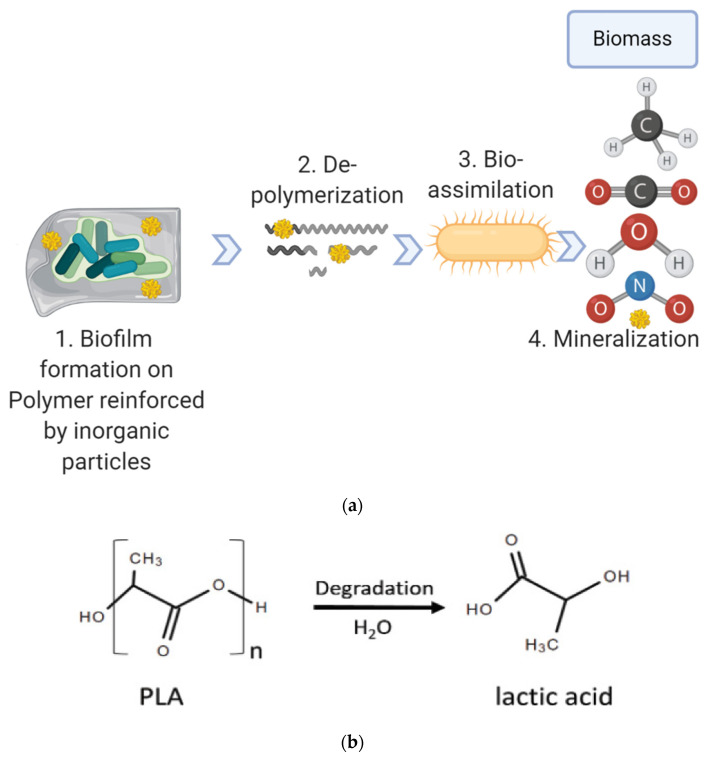
Stepwise biodegradation. (**a**) The whole biodegradation by microorganisms can be outlined in 4 steps: (1) biodeterioration, (2) depolymerisation and mineralisation, (3) bio-assimilation and (4) mineralisation. (**b**) Poly lactic acid (PLA) degrades (hydrolysed) to lactic acid.

The exposure to free environments (sunlight, temperature, humidity, and salinity) and different mechanical stresses plays an important role in the degradation of biodegradable polymers. Biological microflora, including bacteria, fungi, and algae, initiate the colonisation and starting activity to mineralise the biomaterials into carbon dioxide, water, and the biomass. Firstly, microorganisms attach onto the surface of degradable materials and start to form a biofilm. Then, microorganisms of the biofilm secrete extracellular enzymes, which catalyse the depolymerisation of the polymer chain into monomers, dimers, or oligomers. Then, the microorganisms uptake the small molecules and produce primary and secondary metabolites. Finally, those metabolites are mineralised and end products like CO_2_, CH_4_, H_2_O, and N_2_ are formed and released into the environment (Figure 2). Biodegradation can occur under both aerobic and anaerobic conditions.

Bulk or surface erosion mechanisms through hydrolysis are another means of chemical degradation when the polymers contain heteroatoms like esters, anhydrides, amides, or urethanes. PLA has highly hydrolysable ester bonds. Therefore, it can be hydrolysed easily by the above-mentioned process, and it shows compostable properties. However, PLA is less susceptible than other biodegradable polyesters to microbial attacks in the natural environment. Several types of microbes currently isolated from soil or water that can degrade PLA was summarised by Qi et al. [71].

NP reinforcement can exert a significant effect on the biodegradability of the PLA polymer. Therefore, the effect of reinforcing inorganic NPs such as TiO_2_, CuO, ZnO, MgO, and Fe_3_O_4_ in PLA, to synthesise active materials for food packaging and extend the shelf lives of food, on the degradation behaviour of the developed NCs has been widely investigated [72]. In a PLA NC, the NPs act as nucleating agents, thereby controlling the spherulite dimensions of the matrix, which further controls the rate of biodegradation. Better interactions lead to an increase in the amorphous zone and improved degradation of the polymer matrix [73]. It has been established that nanoclays significantly improve PLA biodegradability [74,75]. The biodegradation rate of PLA/TiO_2_ NCs was higher than that of pure PLA, because water molecules can easily penetrate NCs [76]. Further, inorganic NPs such as ZnO act as an accelerator for the hydrolytic degradation of PLA in water specifically below the Tg temperature and specified as heterogeneous catalysts. On the contrary, silanised ZnO NP-reinforced NCs slow down the process of hydrolytic degradation via the delayed diffusion of water molecule into the PLA structure and, therefore, can be used to modify the hydrolytic degradation of PLA-based NCs [77]. Additionally, clay additions also improve the degradability of the PLA matrix. Recently, NCs reinforced with modified Montmorillonite Cloisite 30B depicted an improvement in the PLA biodegradability in an aerobic environment compost, achieving 40% [17].

The degradation study of thymol and Ag-NPs with PLA showed that the inherent biodegradable properties of PLA were improved by the addition of thymol and Ag-NPs, getting a faster degradation rate and meeting the biodegradation legal requirements [40]. The dispersion of TiO_2_ nanofillers in the PLA matrix affected the water absorption and degradation rate. The rapid increase of crystallinity also affected the degradation rate of NCs. Therefore, the hydrolysability of PLA could be controlled by adding TiO_2_ nanoparticles. Nonetheless, these studies suggest that biodegradation is delayed as a result of the enhanced NC barrier properties [76].

## 4. Applications

The incorporation of inorganic NPs is an effective strategy to reduce the permeability and impart antimicrobial properties in PLA; however, their inclusion has also raised health safety concerns associated with the use of metal and inorganic nanoparticles in food packaging applications [72]. In addition to the common bacteriostatic silver nanoparticles, some of them have attracted great interest because of their resistance to rough treatment conditions and the increased inhibition of foodborne pathogens, such as oxidised nanoparticles—TiO_2_, CuO, ZnO, MgO, and Fe_3_O_4_. The research is still ongoing to determine the safety of NCs in food packaging; therefore, the scientific literature on their applications in food systems is still limited. This section will highlight the work that has been carried out on the use of PLA NCs in different food products.

Goh et al. [78] successfully improved the water vapour and oxygen barrier properties of PLA with a sandwich-architectured PLA–graphene composite film consisting of reduced graphene oxide as the core barrier layer. The authors evaluated the potential impact of the improved barrier properties on the shelf lives of edible oil and potato chips using mathematical models that took into account the permeation rate across the films and the lipid oxidation reaction rate. The simulations estimated an eightfold extension of the shelf lives of both nonperishable food products due to the improved barrier. Another approach to improve the oxygen barrier is the use of unmodified montmorillonite clay (Cloisite^®^ Na+) to reinforce PLA. Vilarinho et al. [79] studied the benefits of enhancing the PLA barrier to better protect processed meat products. The lipid oxidation status of sliced salami vacuum packed with NC films containing MMT was monitored during its 90-day shelf life at 5 °C. Packaging with PLA/MMT films successfully reduced the lipid oxidation (lower TBAR and hexanal contents), thus extending the shelf life of sliced salami.

The potential of PLA polymers reinforced with inorganic NPs as active NC food packaging to improve the food safety and extend the shelf lives of perishable food products has also been explored. Several authors have investigated the effect of antimicrobial NC packaging to extend the shelf lives of fresh fruit. For instance, a PLA-based film reinforced with ZnO was explored for fresh-cut apple packaging, which displayed an outstanding result in relation to apple firmness, the total phenolic content, colour, sensory quality, and shelf life [80]. Chi et al. [81] evaluated the capacity of the PLA matrix containing TiO_2_ and Ag for extending the shelf life of mangoes, finding positive effects on the overall acceptability value and freshness of mangoes for a period of 15 days at room temperature. Other authors have investigated the antimicrobial effects in meat and fish products. A storage study of fish (*Otolithes ruber*) fillets packaged in a PLA/ZnO film and stored at a refrigerated temperature for eight days showed a significant reduction in the microbial growth considering the total viable count of the fillet samples. Further, the migration of the Zn^2+^ ions from the PLA/ZnO film after 16 days of storage at refrigeration temperature was reported to be (1.551 mg/100 g of the fillet samples) below the migration limit stated by the National Institute of Health for food contact materials: 40 mg/day on the zinc daily consumption (https://ods.od.nih.gov/factsheets/Zinc-HealthProfessional/ (accessed on 29th March 2021)) [82]. Another study showed the importance of PLA composites (PLA/EO/Ag-Cu) for preserving contaminated fresh chicken and its function as an active packaging during storage for 21 days, wherein the pathogen count was dropped after 15 days of storage in PLA NCs [66]. Longano et al. [83] and Conte et al. [84] proposed to use Cu NPs with PLA for the packaging of dairy products. Conte et al. [84] studied the antimicrobial effects of PLA films embedded with Cu NPs used as packaging materials for dairy products and confirmed that copper nanoparticles exert an antimicrobial effect without sacrificing the key sensory attributes of fiordilatte cheese. Gomashe [85] showed a synergistic effect of gold nanoparticles on active packaging, which is effective against many bacteria.

Other active NC packaging solutions, such as antioxidant materials, can also contribute to improving the quality and extending the shelf lives of food products sensitive to oxidation. Inorganic nanosilver particles embedded with PE composites showed a reduced lipid oxidation of walnuts, hazelnuts, almonds, and pistachios during their shelf lives [86]. Selenium nanoparticles have shown promising results due to their radical scavenging capacities. Multilayer films consisting of PET/adhesive/LDPE-containing SeNPs resulted in the shelf-life extension of hazelnuts, walnuts, and potato chips [87]. Based on previous research, the development of PLA-based antioxidant NCs for shelf-life extension should be explored.

Apart from determining the application of PLA NCs in food products, researchers have also carried out the migration testing of the composites on food stimulants, as outlined in Regulation No. 10/2011 [16]. Vasile et al. [15] studied the migration of three metals (ZnO with bimetallic NPs (Cu and Ag) containing NPs in PLA NC formulations in three different simulant media (distilled water, ethanol 10% (*v/v*) in aqueous solution, and acetic acid 3% (*w/v*) in aqueous solution) for 2 h at 70 °C. The migration of Cu and Zn increased with the amount of ZnO:Cu/Ag NPs in the PLA matrix for all food simulants. They observed that the overall migration for all samples was below 10 mg.dm^−2^ (the accepted value according to Regulation No. 10/2011) [16]. Additionally, PLA reinforced with modified Montmorillonite Cloisite 30B was studied for its stability and suitability as a food contact material. The results revealed that PLA NCs, when in contact with a food simulant, do not produce a strong increase in Al, Ca, Fe, or Mg. Thus, the results indicated that the produced biodegradable NCs are safe and can be in contact with food, according to the commission regulation N.10/2011 [17]. Li et al. [88] studied the migration of TiO_2_ and Ag NPs from PLA NCs to 95% (*v/v*) aqueous ethanol and 3% (*w/v*) aqueous acid food simulant solutions. They observed that the migration rate of PLA/TiO_2_ NCs in an acid simulant solution was significantly higher than an organic solution. It was also established that Ti NPs migration was higher in the first five days, indicating that, initially, NPs were released from the film surface layer, with a subsequent release of NPs from the interior of the NC film. The migration of Ag NPs showed a linear trend during the storage time. The authors stated that the migration ratio of the Ag NPs was higher than the Ti NPs, as Ag NPs have lower particle sizes and higher surface-to-volume ratios. Overall, it was concluded that the NP releases from NC films were within the standard limits.

## 5. Conclusions and Future Directions

Nanomaterial reinforcement in bio-based materials has opened new avenues for green polymers, addressing environmental concerns related with the use of plastics. PLA possesses an accountable thermal processability, which makes it a polymer of choice for food packaging applications; this polymer can, therefore, be processed using injection and blow moulding, extrusion, thermoforming, etc. with respect to other available biopolymers. The use of nanomaterials in a PLA matrix can successfully negate many of the weakness of PLA NCs compared to fossil-based polymers. Metal nanoparticles can improve the mechanical, optical and morphological properties of pristine PLA while contributing to the antimicrobial properties in NC-derived films. It has also been reported that antimicrobial properties can slightly delay the biodegradation process of PLA NCs.

Although much research has been done on the development of PLA-based NCs, their applications in real food packaging systems are limited in the literature. Further research efforts should be done to validate the potential of PLA NCs with added inorganic NPs in real food packaging applications to improve food safety and extend shelf lives, thus contributing to preventing food waste. The authors believe that, in the near future, many fossil-based conventional polymers could be substituted with PLA-based NCs as direct food contact packaging materials.

## Figures and Tables

**Table 3:** Barrier properties of PLA-based NCs reinforced with organic NPs.

NCs	WVTR (Water Vapour Transmission Rate)	OTR (Oxygen Transmission Rate)	Reference
PLA/Ag NPs	Lower WVTR decreased (up to 4%) with addition of 1% Ag NPs underlining the low effect of Ag NPs	1% Ag NPs yielded 22% reduction in OTR value	[55]
PLA/ZnO NPs	NCs reinforced with 9% ZnO decrease WVTR up to 40% while at 15% loading WVTR decreased up to only 20%.	NCs reinforced with 9% ZnO NPs decrease OTR up to 33.5% while at 15% loading OTR value did not decrease.	[57]
PLA/MgO NPs	Reinforcement of MgO NPs increased the WVTR of NCs.	Reinforcement of 1 and 2% MgO NPs reduced OTR around 22 and 25% while no more decrease was observed for 4% MgO reinforced NCs.	[49]
PLA/TiO_2_ and PLA/HNT NPs	The WVTR of NCs loaded with TiO_2_ increased up to loading concentration of 2.5% while it decreases at 5% and 7.5% loading concentration 51 and 47% respectively. For all the selected concentration of HNTs WVTR increased drastically.		[58]

PLA = Poly lactic acid; TiO_2_ = Titanium dioxide; ZnO = Zinc oxide; MgO = Magnesium oxide; Ag = Silver; HNT = Halloysite nanotubes; NPs = Nanopartilces.

## Data Availability

Not applicable.
